# Artificial intelligence-based approaches for monitoring medication adherence among cardiovascular disease patients: a scoping review

**DOI:** 10.1186/s12911-026-03422-3

**Published:** 2026-03-13

**Authors:** Nishit Kumar Bebarta, Anusree Prabhakaran, Abhinav Bhat, P. Sudhakara Upadya, U. Shashikiran, Satyanarayana Poojari, Arathi P. Rao, Anil K. Bhat, Elstin Anbu Raj S

**Affiliations:** 1 Department of Global Public Health Policy and Governance, Prasanna School of Public Health, Manipal Academy of Higher Education, Manipal, 576104 India; 2 Department of Computer Science and Engineering, Manipal Institute of Technology, Manipal Academy of Higher Education, Manipal, 576104 India; 3 Manipal School of Information Science, Manipal Academy of Higher Education, Manipal, 576104 India; 4 Department of General Medicine, Dr. TMA Pai Hospital, Udupi, Manipal Academy of Higher Education, Manipal, 576104 India; 5 Department of Applied Statistics and Data Science, Prasanna School of Public Health, Manipal Academy of Higher Education, Manipal, 576104 India; 6 Department of Hand surgery, Kasturba Medical College, Manipal Academy of Higher Education, Manipal, 576104 India; 7 Department of Health Technology and Informatics, Prasanna School of Public Health, Manipal Academy of Higher Education, Manipal, 576104 India

**Keywords:** Cardiovascular diseases, Medication adherence, Artificial intelligence, Machine learning, Digital health, mHealth, Internet of Things (IoT), Scoping review

## Abstract

**Background:**

Cardiovascular diseases (CVDs) are still a leading cause of death worldwide, and the impact of the disease in lower-income countries is dire. Patients must take medications on a regular basis for effective management of CVD; however, the level of efficacious self-management is far below what is required. Artificial Intelligence (AI) shows promise in evaluating different data sets, aiding in clinical decision processes, and facilitating active engagement in the self-management of medication. The purpose of this scoping review was to analyse the available literature on the approaches that have been employed in using AI to improve medication adherence in patients with CVD.

**Methods:**

This research was done according to the PRISMA-ScR guidelines. A comprehensive literature search was conducted on June 19, 2025, across PubMed, Embase, CINAHL, Scopus, and Web of Science using pre-established eligibility standards. Screening was done using Rayyan across two stages. Data was extracted on the characteristics of the study, AI-based approaches and their methods, measures of adherence, and the outcomes reported.

**Results:**

Sixteen studies were included based on the inclusion criteria. The included studies varied in setting, sample size, and disease conditions. AI-based approaches comprised natural language processing, reinforcement learning, and machine learning algorithms such as neural networks, random forests, support vector machines, and decision tree models. Interventions were delivered through smartphone applications, clinical workflow systems, Internet of Things (IoT) enabled devices, and chatbots. Medication adherence was assessed using pharmacy refill data, electronic health records (EHR), validated surveys, device-based monitoring, administrative claims, and biochemical testing. Several AI-based approaches showed improvements in adherence; however, across studies, reports were inconsistent, and methodological variation limited comparability. Evidence from low- and middle-income countries remained limited.

**Conclusion:**

AI-based approaches have been applied to monitor and support medication adherence among patients with CVD; however, the outcome measures are inconsistent due to limited population diversity and variable study quality, restricting the comparability across existing evidence. Standardized adherence metrics, rigorous methodologies, and broader evaluations in diverse settings are essential to strengthen future research and support real-world decisions.

**Supplementary Information:**

The online version contains supplementary material available at 10.1186/s12911-026-03422-3.

## Introduction

Globally, cardiovascular diseases (CVDs) are one of the leading causes of death, accounting for 18 to 20 million deaths every year [1, 2]. In 2022, approximately 19.8 million, which is about one-third of all deaths in the world, were due to CVDs. Heart attacks and strokes contribute to four out of every five cases, and nearly one-third occur before the age of 70 years [1, 3]. Over three-quarters of these deaths occur in nations belonging to the low- and middle-income category [2, 3]. By 2023, an estimated 620 million people were known to be living with CVDs, with 60 million new cases annually and projections showing a 90% rise by 2050 [4]. CVDs encompass coronary heart disease, cerebrovascular disease, rheumatic heart disease, and other vascular conditions [1].

Management of CVD hinges on taking medications as prescribed. Medication adherence based on the prescription can greatly help in improving blood pressure control, regulating cholesterol, stabilizing blood sugar, and decreasing the risk of adverse cardiovascular events [5]. Improved medication adherence results in fewer hospitalizations, decreased economic burden, and lower mortality, demonstrating its importance in the long-term control of CVD [6]. After many years of targeting CVD and attempting to educate and incorporate technology focusing on improving medication adherence among patients, these interventions have had minimal impact on the improvement of CVD outcomes [7, 8].

Digital tools like health apps and fitness trackers are now more common in the management of CVD. Even though they collect constant information, poor links to doctor routines can reduce their usability [9, 10]. AI techniques such as natural language processing, neural networks, and decision tree algorithms can help to analyze this vast information, spot key trends of disease, guide health care providers toward better-informed decision making, and help in monitoring medication adherence [6–8].

Though literature exists that examines the role of AI in medication adherence among CVDs, most of these depend on electronic health record (EHR) data to analyze adherence patterns and predict future behavior [5, 11]. A few studies have reported the development of AI-based technologies, while others have explored applications such as mobile health interventions, chatbots, and predictive models, including natural language processing, decision trees, and neural networks [7, 12]. Although reviews on AI in chronic diseases exist, there is a lack of comprehensive work focusing on AI-based approaches for medication adherence in CVD. A scoping review was thus found suitable for mapping the existing evidence, offering an overview of the extent of research done on AI-based approaches, highlighting the gaps to guide future studies.

## Objective

The scoping review aimed to provide an overview of research on AI-based approaches for monitoring medication adherence among patients with CVDs.

## Methods

The scoping review was conducted based on the JBI scoping review guidelines [13], and was reported based on the Preferred Reporting Items for Systematic Reviews and Meta-Analyses extension for Scoping Reviews (PRISMA-ScR) guidelines [14], and provided in supplementary file 1. The protocol was registered in Open Science Framework (OSF) (10.17605/OSF.IO/MK6H7).

### Search methodology

On June 19th, 2025, an extensive search across five databases, including PubMed, Embase, CINAHL, Scopus, and Web of Science, was performed, employing search strategies formulated using the Population, Concept, and Context (PCC) framework. The Medical Subject Headings (MeSH) terms and suitable keywords like cardiovascular diseases, artificial intelligence, Large Language Models, and medication adherence were identified based on the Population, Concept, and Context. The terms were connected using Boolean operators for developing efficient search strategies.

We included peer-reviewed articles without any restrictions on the publication date. Grey literature sources such as dissertations, conference proceedings, or reports, and non-English language papers were excluded.

The search strategy was constructed using the PCC framework. CVD-related terms were connected with the terms of AI methods and applied across the global context. Boolean operators (AND/OR) were used to integrate these concepts in order to find pertinent observational and interventional studies. NKB developed the search strategy and validated it with EARS, who is an expert in scoping review methodology. The search strategy and PRISMA–S checklist is provided in Supplementary file 2 and 3 respectively.

### Study selection

The inclusion criteria of this review were developed based on the PCC framework as presented in Table 1.

**Table 1:** Inclusion criteria

Category	Inclusion Criteria
Population	Studies focused on CVDs, including WHO-classified conditions: deep vein thrombosis/pulmonary embolism, congenital heart disease, peripheral arterial disease, rheumatic heart disease, coronary heart disease, and cerebrovascular disease.
Concept	Studies that used AI-based approaches for monitoring medication adherence such as models, systems, platforms to assess, predict, track or influence medication adherence behavior
Context	Global
Study Design	Original research articles with interventional or observational study design

### Screening process

All the articles retrieved from the five databases were uploaded to Rayyan (www.rayyan.ai). Duplicates were identified and removed. After deduplication, the remaining studies were screened in two stages (title and abstract screening and full-text screening) by two separate reviewers, NKB and AP. Based on the inclusion criteria, the title and abstract were screened, and the selected studies were considered for full-text screening.

In case of conflicts, they were resolved by discussion between the two reviewers and APR was consulted using a consensus building approach.

### Data charting

NKB, AP, and AB independently extracted data using a pre-defined and validated data extraction sheet developed in Microsoft Excel. Data included key variables, such as author names, year of publication, country of origin, study setting, sample size, gender, disease condition, AI-based approach, methods, validity, sensitivity, dataset, tool used to measure adherence, and improvement in adherence. In this review, “validity” referred to whether the included studies provided official validation of the AI-based strategy, and “sensitivity” referred to whether the AI model’s or tool’s sensitivity or relevant performance measures have been revealed. Any conflicts were first resolved through discussion between the three reviewers, and if needed, other authors were consulted.

### Data synthesis and reporting

The research-related attributes focused on key aspects relevant to AI-based approaches for medication adherence, covering the following elements: (i) different AI-based approaches and their methods used, (ii) dataset characteristics for the pilot or real-world test, (iii) validity and sensitivity of the tool, (iv) tool used to measure adherence, and (v) improvement in medication adherence outcomes. The results were synthesized using suitable figures and tables wherever applicable.

## Results

**Fig. 1:**
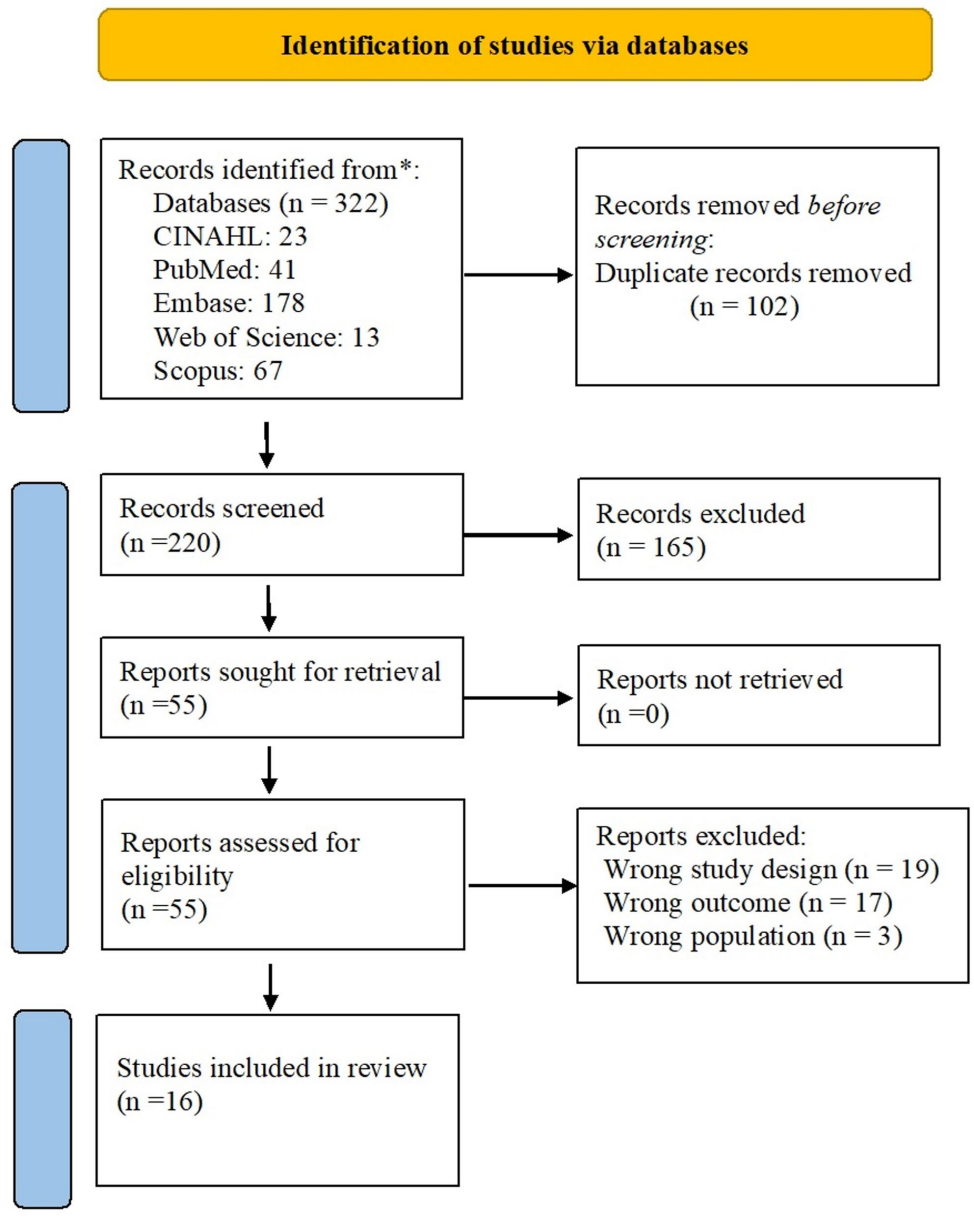
PRISMA Flow diagram of the study selection

In total, 322 studies were retrieved (CINAHL: 23, PubMed: 41, Embase: 178, Web of Science: 13, Scopus: 67). Of these, 102 studies were duplicates. After deleting the duplicates, 220 studies were screened for title and abstract. After title/abstract screening, 165 studies were excluded, and the full text of the 55 included studies was screened. After full-text screening, 38 articles were excluded due to incorrect study design (*n* = 19), inappropriate outcome (*n* = 17), or ineligible population (*n* = 3). A total of 16 studies were included in this scoping review. The screening process for including studies is illustrated in Fig. 1 using a PRISMA flow diagram.

### Study characteristics

**Fig. 2:**
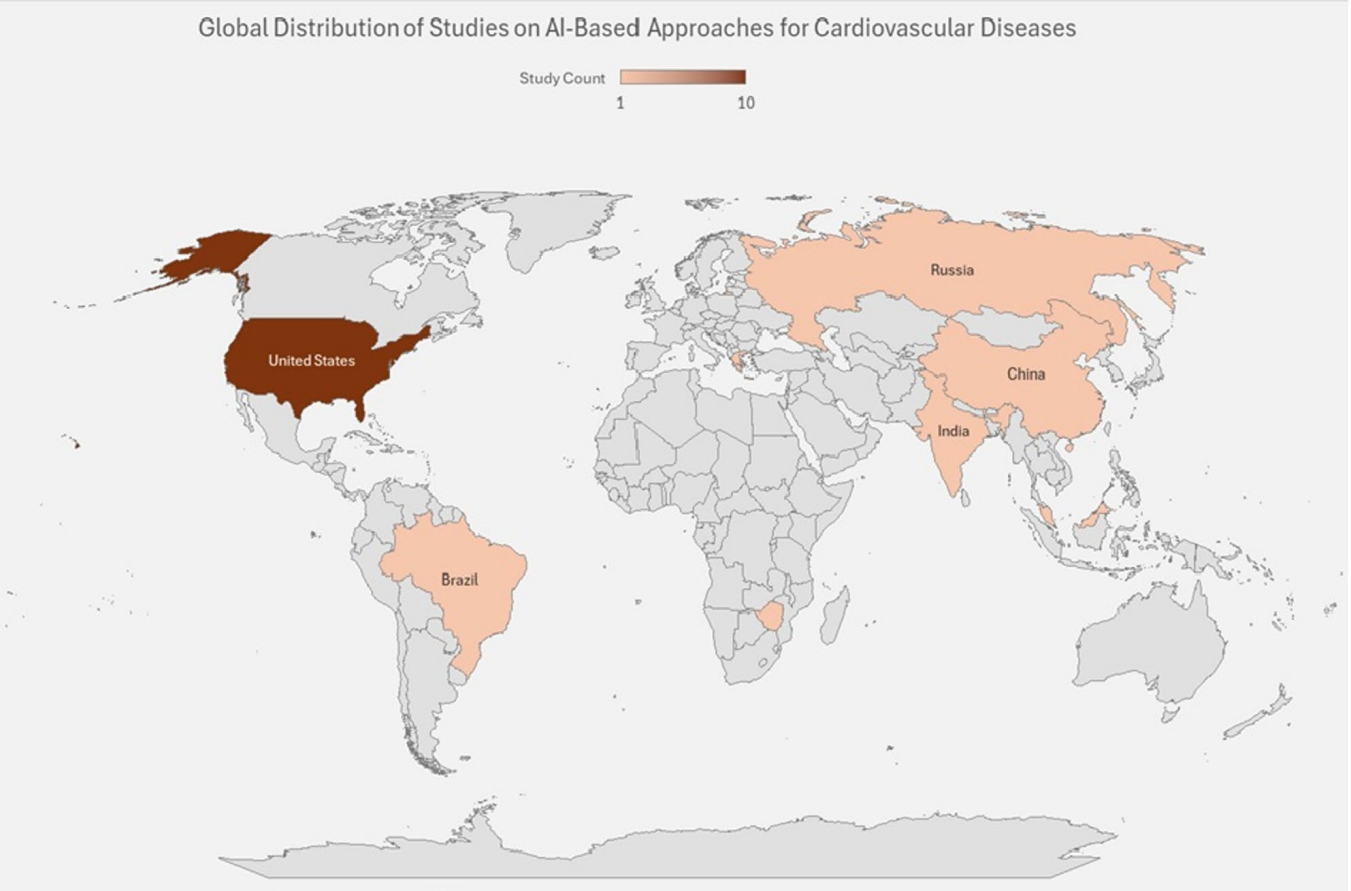
Geographical distribution of the included studies

In the included 16 studies, the majority were conducted in the United States [15–23], and the remaining studies were from Brazil [24], Zimbabwe [25], India [26], China [27], Russia [28], Malaysia [29], and Greece [30], as shown in Fig. 2. The number of participants in the studies varies from 28 to over 500,000 participants and more. The study populations mainly included patients with CVDs, including heart failure [15, 21], hypertension [17, 19, 20, 24, 25], coronary artery disease [16, 20, 21, 28], diabetes [18, 20, 25, 26], and other related comorbidities. Gender representation varied across studies; some reported mixed male-female participation, whereas others did not report. The included studies were conducted across diverse settings, including clinical settings [15, 18, 19, 22], community based [16, 17, 21], and digital health environments [20, 23, 25, 26, 29]. A detailed overview of the extracted study is presented in Table 2.

**Table 2:** Overview of the extracted study characteristics

Author and year	Study setting	sample size	Gender	Disease Conditions	AI-Based Approach (Model / System / Platform)	Methods	Validity reported	Sensitivity reported	Dataset for validity and sensitivity	Tool used to measure adherence	Adherence improvement
Topaz et al., 2017	USA	8901	Male (57%), Female (48.8%)	Heart failure	MTERMS (Medical Text Extraction, Reasoning and Mapping System)	Natural Language Processing (NLP)	YES	YES	300	Electronic discharge summaries	NO
Horne et al., 2022	USA	186	Male (74.2%), Female (25.8%)	Coronary disease	SARSA (State-Action-Reward-State-Action)	Reinforcement Learning (RL)	NR	NR	NR	Proportion of days covered	YES
Li et al., 2019	USA	10,000	NR	Hypertension	Exhaustive CHAID (Chi-squared Automatic Interaction Detection)	Machine Learning (ML)	YES	NR	10,000	Self-reported survey	NO
da Silva et al., 2019	Brazil	100	NR	Hypertension	Custom-built intelligent system integrated with IoT and a smart medicine cabinet	Decision tree algorithms	NR	NR	NR	Commercial electronic devices	NO
Worrall et al., 2025	USA	10,477	NR	Hypertension, Hyperlipidemia, Diabetes mellitus	Proprietary clinical workflow management system	Predictive analytics	NR	NR	NR	Pharmacy claims data	YES
Persell et al., 2018	USA	300	NR	Hypertension	Hypertension Personal Control Program (HPCP) app	NR	NR	NR	NR	Self-report via HPCP app	NO
Ho et al., 2025	USA	9501	NR	Hypertension, Hyperlipidemia, Diabetes, Coronary artery disease, Atrial fibrillation	Fixed message chatbot	NR	NR	YES	NR	Pharmacy refill data using Proportion of Days Covered	YES
Kanyongo et al., 2024	Zimbabwe	8141	Male (41%), Female (59%)	Diabetes, Hypertension	NR	Machine Learning (ML)	YES	YES	8141	Medication refill from CIMAS Transactional Processing System (TPS)	NO
Ruksakulpiwat et al., 2024	USA	326	Male (46%), Female (54%)	Heart attack, Coronary artery disease, Angina, congestive heart failure	NR	Decision Tree	NR	NR	NR	Health and Retirement Study 2016 Nonadherence module	NO
Feng et al., 2024	India	NR	NR	Diabetes, Hypertension	NR	Neural Networks	YES	YES	NR	Data from Electronic health records, wearables, mobile apps	YES
Marrs et al., 2023	USA	6375	Male (52.2%), Female (47.8%)	Hypertension, hyperlipidemia, Diabetes, Coronary artery disease, Atrial fibrillation	NR	AI chatbot algorithm	NR	NR	NR	Pharmacy refill data	NO
Xiao et al., 2022	China	408	Male (77.7%), Female (22.3%)	Acute myocardial infarction	NR	Machine Learning (ML)	YES	NR	408	Clinical records	NO
Aziz et al., 2020	Malaysia	160	Male (70.6%), Female (29.4%)	Hypertension	NR	Artificial Neural Network (ANN)	YES	YES	160	Malaysian Medication Adherence Scale	NO
Labovitz et al., 2017	USA	28	Male (47%), Female (53%)	Ischemic stroke	AiCure AI Platform	Neural networks	NR	NR	NR	Visual confirmation, pill counts, plasma drug concentration levels	YES
Karanasiou et al., 2016	Greece	90	NR	Heart failure	NR	Supervised machine learning classification algorithms	YES	YES	90	Clinician estimation	NO
Zakeri et al., 2021	Russia	269	Male (51%), Female (49%)	Acute myocardial infarction, Angina pectoris	NR	Gradient boosting model	YES	YES	269	Self-reported Questionnaire	NO

NR = Not Reported in the study

### Types of AI-based approaches and methods

**Fig. 3:**
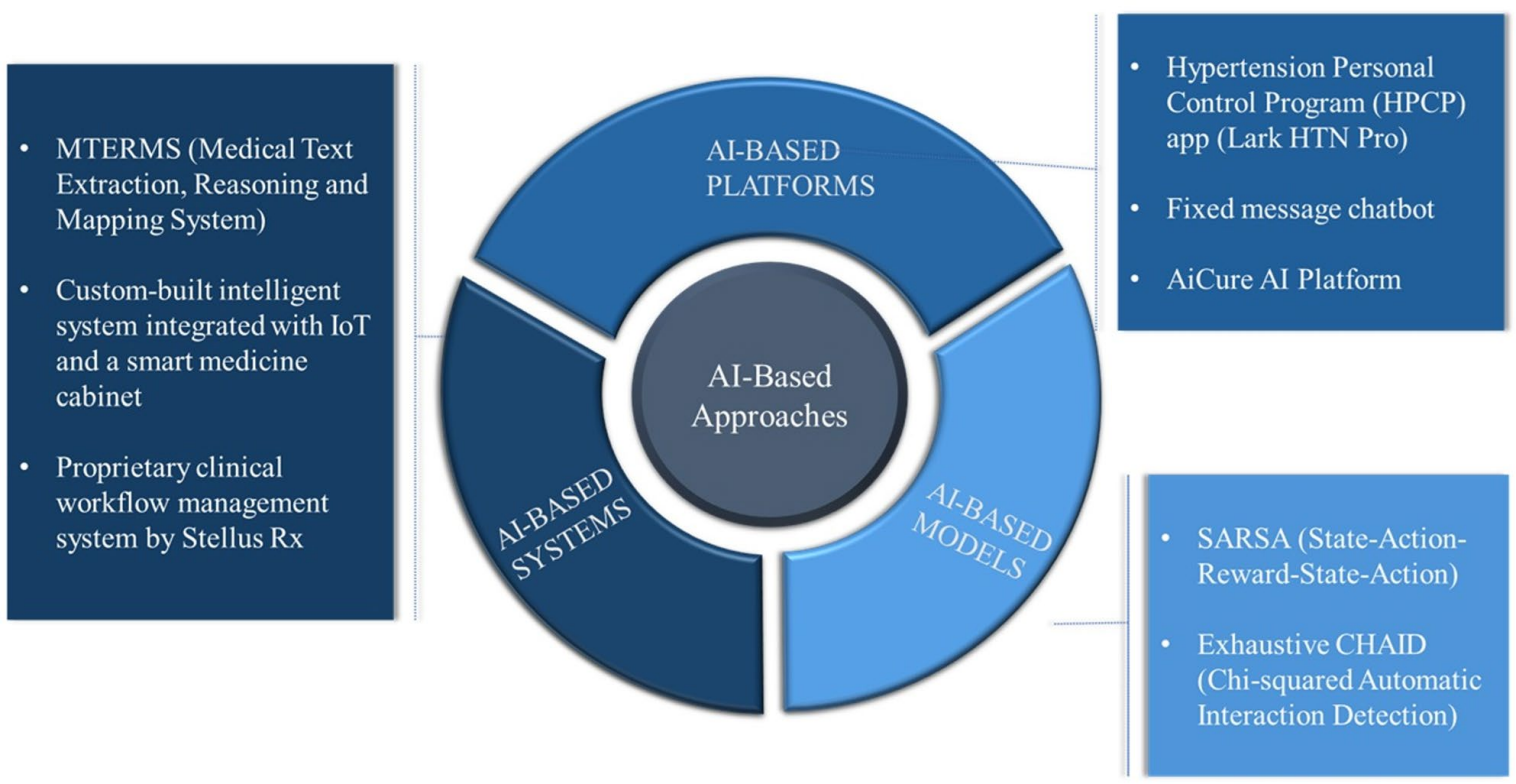
Classification of AI-based approaches and their methods

**Fig. 4:**
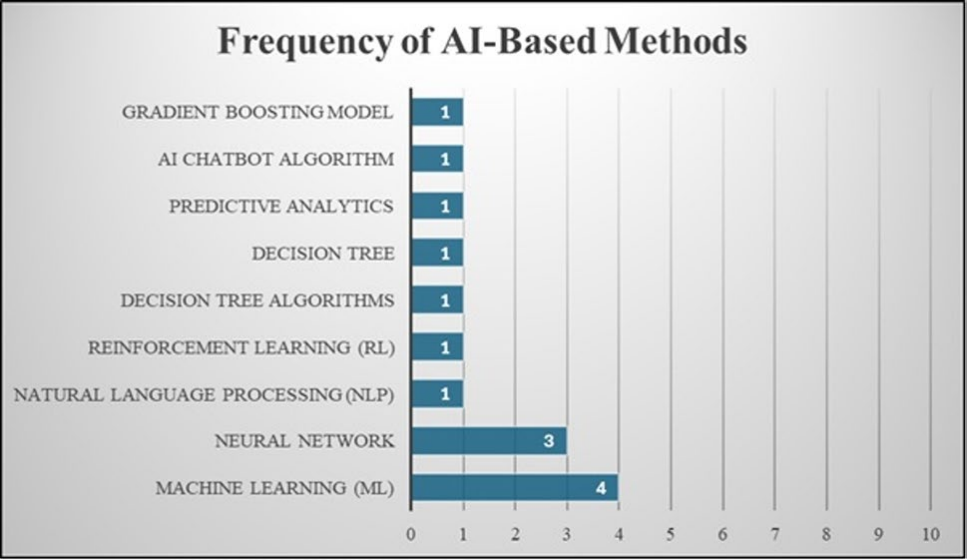
The frequency of AI-based methods used in the studies

The studies included in this review consist of a wide range of AI-based approaches and their methods. AI-based approaches were classified into three categories: [1] AI-based models; [2] AI-based systems; and [3] AI-based platforms, as shown in Fig. 3. The methods used in the approaches were natural language processing (NLP) [15], reinforcement learning [16], multiple machine learning (ML) algorithms, random forests, neural networks, support vector machines [25], and gradient boosting models [28]. Several studies employed decision tree classifiers [21, 25, 27, 30, 31] and chatbot systems delivering preprogrammed messages [20, 22]. The frequency of AI-based methods used in the studies is shown in Fig. 4. The main objectives of these AI-based approaches included predictive analytics, medication adherence behaviors classification, ingestion verification using computer vision, and improved management of adherence-related data [17, 18, 31].

### Adherence measurement and tools

The included studies assessed medication adherence using a variety of data sources and evaluation procedures. Adherence was assessed using EHR [15], pharmaceutical refill data [16], validated self-report surveys [17], device-generated data [23, 26, 31], administrative claims, and biochemical testing.

Selected studies reported AI-supported adherence assessment. NLP was utilized to extract adherence data from EHR [15]. Pharmacy refill records were evaluated to determine the proportion of days covered (PDC) [16]. In population-based samples, self-reported adherence was assessed using validated surveys [17].

Several studies used real-time adherence data gathered from commercial electronic devices, including mobile devices, wearables, and smart medicine cabinets [23, 26, 31]. In clinical program settings, administrative data sources such as pharmaceutical claims and Medicare Star ratings were employed to track adherence [18, 20, 25]. Additional adherence measuring approaches included clinician estimation [30], text message-based refill tracking combined with chatbot interactions [20], and biochemical tests in specific study circumstances [23].

### Effectiveness of AI-based approaches

Several AI-based approaches for medication adherence showed improvements in adherence outcomes, the measurements and reporting methods differed between studies. In a study of 186 participants (n), reinforcement learning-based guided behavioral nudges reported 10.3% increase in medication adherence, as measured by the PDC [16]. A pharmacy-led, AI-supported adherence program reported improvements across multiple chronic conditions, including a 5.9% increase in hypertension, 7.9% in cholesterol, and 6.4% in diabetes medication adherence, based on pharmacy claims-derived adherence measures in a large clinical population (*n* = 10,477) [18]. While several studies with digital AI-based approaches reported positive adherence results, some studies did not provide specific quantitative adherence estimates. Baseline values, follow-up time periods, and comparator designs were provided inconsistently between studies, making direct comparisons of adherence results difficult.

## Discussion

This scoping review collected evidence from 16 studies focusing on AI-based approaches for improving medication adherence in CVD populations across multiple health systems. These approaches consist of a wide range of AI methods, including NLP [15, 26], reinforcement learning [16], and ML algorithms, provided through platforms such as applications, workflow management systems, and IoT-enabled devices [17, 18, 31].

Medication adherence measurement methods ranged widely, including refill records of pharmacy [20, 22, 25], EHR [15, 26], self-reports [17, 21], device-based monitoring [23, 31], administrative claims [18], and biochemical testing [23]. Studies showed mixed effectiveness outcomes but generally positive, such as reinforcement learning-based interventions improved adherence by 10.3% [16]. Despite these positive findings, gaps persisted in longitudinal follow-up, standardization in adherence metrics, diversity in populations, and validation of AI models [17, 32].

Findings from this review line up with recent systematic reviews and meta-analyses showing that AI-based approaches have the potential to improve medication adherence in chronic disease populations. Reis et al. (2025) reported that randomized trials using AI tools such as cognitive-behavioral AI coaching and real-time video monitoring achieved improvements in medication adherence ranging from 6.7% to 32.7% [33]. Similarly, Zavaleta-Monestel et al. (2025) found that smart devices such as app, smart watch and mobile applications enhanced medication adherence, though the lack of standardized measurement approaches remains a concern (41). These findings remain consistent with improvement rates observed for reinforcement learning nudges [16] and predictive analytics interventions in the present review [18].

However, studies emphasized that persistent challenges in achieving consistent clinical benefits. Bhagavathula et al. (2023) reported wide variability in eHealth interventions for cardiovascular medication adherence after acute coronary syndrome, with results showing fewer benefits than standard care [35]. This supports our review’s results regarding variability in the measurement of medication adherence and the need to standardize outcome measures. Shuja et al. (2025) further showed that despite technological improvements, real-world integration into clinical workflows remains limited [36].

Studies with a small scale, such as Babel et al. (2021), identified that a substantial gain in medication adherence (67%) was achieved using smartphone apps with a neural network for ingestion verification [37]. These findings reflecting the importance of similar AI-enabled adherence verification tools described in this review [23]. Issues with methodology like inconsistent medication adherence tools and limited evaluation of user engagement and transparency of models, were emphasized in Kanyongo et al. (2023) [38].

This scoping review shows that AI-based approaches like predictive analytics, behavioral nudges, conversational agents, and ingestion verification tools have major potential for medication adherence improvement among CVD patients. These approaches provide opportunities to deliver automated support at a large scale, reduce workload in clinical settings, and provide more personalized patient care [16, 18].

Although this scoping review did not conduct a systematic risk-of-bias assessment, notable patterns in research design and reporting quality were found throughout the included studies. The majority of research used observational or program evaluation designs, with only a few using explicit comparison groups or controlled study designs [16, 18]. Follow-up times and baseline adherence values were inconsistently reported, reducing the interpretation of reported percentage changes in adherence outcomes. There was also significant variation in adherence assessment methodologies, including pharmacy refill data, EHR, self-reported survey responses, device-based monitoring, and administrative claims [15–18, 23]. Additionally, the reporting of AI-related components differed across studies, with some studies providing minimal information on model validation, performance metrics, and handling missing data [15, 16, 18, 32]. These variables should be considered when evaluating reported adherence outcomes, emphasizing the need for more uniform and transparent reporting in future AI-based drug adherence research.

For practical implementation, the successful adoption of AI-based approaches in healthcare settings will require standardization in adherence measurement methods, privacy of both health care providers and patients, security, and ethical considerations [18, 32].

### Limitations

This scoping review identified several limitations, such as inconsistency in adherence improvement metrics and diversity in adherence measurement tools, causing limited direct comparability across studies. Longitudinal data was limited, causing restricted insights into the sustained effects of AI-based approaches. Population diversity was also limited, with less focus on low- and middle-income country settings. AI models’ validity and sensitivity processes were inconsistent, and few studies examined user integration of AI-based approaches into routine clinical practice [15, 16, 18].

### Future directions

- Standardizing adherence outcomes to enable consistent comparisons across studies.
- Assessing long-term effectiveness of AI-based approaches to determine sustained benefits for adherence and clinical outcomes.
- Expanding research in low- and middle-income countries to enhance global applicability and equity.
- Evaluating engagement, workflow feasibility, and cost-effectiveness to support real-world integration.
- Improving transparency in AI model development, including reporting of validation methods, risk assessments, and performance metrics, to build trust and regulatory acceptance.
- Exploring multimodal and personalized AI-based approaches combining behavioral science, advanced analytics, and real-time monitoring for tailored patient support.

## Conclusion

This scoping review highlights the increasing use of AI-based approaches to support medication adherence among patients with CVDs. While studies reported improvements in adherence-related outcomes, the overall evidence is constrained by limited analytical depth, heterogeneous adherence measurement methods, and variability in study populations and reporting practices, which restricts meaningful synthesis and comparison across studies.

AI-assisted adherence systems demonstrate potential to enhance patient engagement, support clinical workflows, and enable scalable digital health solutions. However, the effective translation of these approaches into routine practice requires standardized outcome measures, more rigorous and transparent methodologies, and broader evaluation across underrepresented settings. Future research should prioritize designs that align with real-world healthcare workflows and assess long-term integration and sustainability to strengthen the evidence base and inform clinical and policy decision-making.

## Supplementary Information

Below is the link to the electronic supplementary material.

Supplementary Material 1: PRISMA ScR checklist for the scoping review

Supplementary Material 2: Search strategy for each database

Supplementary Material 3: PRISMA S checklist

## Data Availability

All the data used in this study is from publicly available literature.

## Abbreviations

AI: Artificial Intelligence
CVDs: Cardiovascular diseases
EHR: Electronic health record
IoT: Internet of Things
JBI: Joanna Briggs Institute
LASI: Longitudinal Ageing Study in India
LLMs: Large Language Models (appears in Methods but spelled out in keywords)
MAHE: Manipal Academy of Higher Education
MeSH: Medical Subject Headings
ML: Machine learning
mHealth: Mobile health
NLP: Natural language processing
OSF: Open Science Framework
PCC: Population, Concept, Context
PDC: Proportion of days covered
PRISMA-ScR: Preferred Reporting Items for Systematic Reviews and Meta-Analyses extension for Scoping Reviews
