# Supplementary material for: Artificial intelligence-based approaches for monitoring medication adherence among cardiovascular disease patients: a scoping review
Source: BMC Med Inform Decis Mak. 2026 Mar 13;26:132. doi: 10.1186/s12911-026-03422-3 (PMC13101352; doi:10.1186/s12911-026-03422-3)
Supplement: Supplementary file 2 — Supplementary Material 2: Search strategy for each database [file 12911_2026_3422_MOESM2_ESM.pdf]

PubMed: 41

((("artificial intelligence"[MeSH Terms] OR Artificial Intelligence[Text Word] OR Computer Reasoning[Text Word] OR Machine Intelligence[Text Word] OR Computational Intelligence[Text Word] OR Computer Vision Systems[Text Word] OR Knowledge Acquisition[Text Word] OR "Machine learning"[Text Word] OR Transfer Learning[Text Word] OR "Large Language Model"[Text Word] OR Adaptive Algorithm[Text Word] OR Trend Analysis[Text Word] OR "Machine Pattern Recognition"[Text Word] OR "Machine Prediction Methods"[Text Word])) AND (("medication adherence"[MeSH Terms] OR "medication adherence"[Text Word] OR "drug adherence"[Text Word] OR "prescription adherence"[Text Word] OR "medication persistence"[Text Word] OR "medication compliance"[Text Word] OR "drug compliance"[Text Word]))) AND (("cardiovascular diseases"[MeSH Terms] OR "cardiovascular diseases"[Text Word] OR "adverse cardiac event"[Text Word] OR ("coronary disease"[MeSH Terms] OR "coronary disease"[Text Word] OR "coronary heart disease"[Text Word]) OR ("cerebrovascular disorders"[MeSH Terms] OR "cerebrovascular disorders"[Text Word] OR "brain vascular disorders"[Text Word] OR "intracranial vascular disorders"[Text Word] OR "cerebrovascular insufficiency"[Text Word] OR "cerebrovascular occlusion"[Text Word]) OR ("peripheral arterial disease"[MeSH Terms] OR "peripheral arterial disease"[Text Word]) OR ("rheumatic heart disease"[MeSH Terms] OR "rheumatic heart disease"[Text Word] OR "bouillaud disease"[Text Word]) OR ("heart defects, congenital"[MeSH Terms] OR "congenital heart disease"[Text Word] OR "abnormality heart"[Text Word] OR "malformation of heart"[Text Word]) OR ("venous thrombosis"[MeSH Terms] OR "deep vein thrombosis"[Text Word] OR ("venous thrombosis"[MeSH Terms] OR ("Venous"[All Fields] AND "Thrombosis"[All Fields]) OR "venous thrombosis"[All Fields] OR "phlebothrombosis"[All Fields]) OR "Phlebothromboses"[Text Word] OR "thrombosis venous"[Text Word]) OR ("pulmonary embolism"[MeSH Terms] OR "pulmonary embolism"[Text Word] OR "pulmonary thromboembolisms"[Text Word]))))

EMBASE: 178

(('artificial intelligence'/exp OR 'artificial intelligence':ti,ab,kw OR 'computer reasoning':ti,ab,kw OR 'machine intelligence':ti,ab,kw OR 'computational intelligence':ti,ab,kw OR 'computer vision systems':ti,ab,kw OR 'knowledge acquisition':ti,ab,kw OR 'machine learning':ti,ab,kw OR 'transfer learning':ti,ab,kw OR 'large language model':ti,ab,kw OR 'adaptive algorithm':ti,ab,kw OR 'trend analysis':ti,ab,kw OR 'machine pattern recognition':ti,ab,kw OR 'machine prediction methods':ti,ab,kw) AND ('cardiovascular disease'/exp OR 'cardiovascular diseases':ti,ab,kw OR 'adverse cardiac event':ti,ab,kw OR ('coronary artery disease'/exp OR 'coronary disease':ti,ab,kw OR 'oronary heart disease':ti,ab,kw) OR ('cerebrovascular disease'/exp OR 'cerebrovascular disorders':ti,ab,kw OR 'brain vascular disorders':ti,ab,kw OR 'intracranial vascular disorders':ti,ab,kw OR 'cerebrovascular insufficiency':ti,ab,kw OR 'cerebrovascular occlusion':ti,ab,kw) OR ('peripheral arterial disease'/exp OR 'peripheral arterial disease':ti,ab,kw) OR ('rheumatic heart disease'/exp OR 'rheumatic heart disease':ti,ab,kw OR 'bouillaud disease':ti,ab,kw) OR ('congenital heart malformation'/exp OR 'congenital heart disease':ti,ab,kw OR 'abnormality heart':ti,ab,kw OR 'malformation of heart':ti,ab,kw) OR ('vein thrombosis'/exp OR 'deep vein thrombosis':ti,ab,kw OR ('vein thrombosis'/exp OR ('venous' AND 'thrombosis') OR 'venous thrombosis' OR 'phlebothrombosis') OR 'phlebothromboses':ti,ab,kw OR 'thrombosis venous':ti,ab,kw) OR ('lung embolism'/exp OR 'pulmonary embolism':ti,ab,kw OR 'pulmonary thromboembolisms':ti,ab,kw))) AND ('medication compliance'/exp OR 'medication adherence':ti,ab,kw OR 'drug adherence':ti,ab,kw OR 'prescription

adherence':ti,ab,kw OR 'medication persistence':ti,ab,kw OR 'medication compliance':ti,ab,kw OR 'drug compliance':ti,ab,kw)

Scopus: 67

(((INDEXTERMS ("artificial intelligence") OR TITLE-ABS-KEY ("Artificial Intelligence") OR TITLE-ABS-KEY ("Computer Reasoning") OR TITLE-ABS-KEY ("Machine Intelligence") OR TITLE-ABS-KEY ("Computational Intelligence") OR TITLE-ABS-KEY ("Computer Vision Systems") OR TITLE-ABS-KEY ("Knowledge Acquisition") OR TITLE-ABS-KEY ("Machine learning") OR TITLE-ABS-KEY ("Transfer Learning") OR TITLE-ABS-KEY ("Large Language Model") OR TITLE-ABS-KEY ("Adaptive Algorithm") OR TITLE-ABS-KEY ("Trend Analysis") OR TITLE-ABS-KEY ("Machine Pattern Recognition") OR TITLE-ABS-KEY ("Machine Prediction Methods"))) AND ((INDEXTERMS ("medication adherence") OR TITLE-ABS-KEY ("medication adherence") OR TITLE-ABS-KEY ("drug adherence") OR TITLE-ABS-KEY ("prescription adherence") OR TITLE-ABS-KEY ("medication persistence") OR TITLE-ABS-KEY ("medication compliance") OR TITLE-ABS-KEY ("drug compliance")))) AND ((INDEXTERMS ("cardiovascular diseases") OR TITLE-ABS-KEY ("cardiovascular diseases") OR TITLE-ABS-KEY ("adverse cardiac event") OR (INDEXTERMS ("coronary disease") OR TITLE-ABS-KEY ("coronary disease") OR TITLE-ABS-KEY ("coronary heart disease")) OR (INDEXTERMS ("cerebrovascular disorders") OR TITLE-ABS-KEY ("cerebrovascular disorders") OR TITLE-ABS-KEY ("brain vascular disorders") OR TITLE-ABS-KEY ("intracranial vascular disorders") OR TITLE-ABS-KEY ("cerebrovascular insufficiency") OR TITLE-ABS-KEY ("cerebrovascular occlusion")) OR (INDEXTERMS ("peripheral arterial disease") OR TITLE-ABS-KEY ("peripheral arterial disease")) OR (INDEXTERMS ("rheumatic heart disease") OR TITLE-ABS-KEY ("rheumatic heart disease") OR TITLE-ABS-KEY ("bouillaud disease")) OR (INDEXTERMS ("heart defects, congenital") OR TITLE-ABS-KEY ("congenital heart disease") OR TITLE-ABS-KEY ("abnormality heart") OR TITLE-ABS-KEY ("malformation of heart")) OR (INDEXTERMS ("venous thrombosis") OR TITLE-ABS-KEY ("deep vein thrombosis") OR (INDEXTERMS ("venous thrombosis") OR (ALL (venous) AND ALL (thrombosis)) OR ALL ("venous thrombosis") OR ALL (phlebothrombosis)) OR TITLE-ABS-KEY (phlebothromboses) OR TITLE-ABS-KEY ("thrombosis venous")) OR (INDEXTERMS ("pulmonary embolism") OR TITLE-ABS-KEY ("pulmonary embolism") OR TITLE-ABS-KEY ("pulmonary thromboembolisms"))))) AND (LIMIT-TO (LANGUAGE , "English"))

CINAHL: 23

((((MH "artificial intelligence+") OR "Artificial Intelligence" OR "Computer Reasoning" OR "Machine Intelligence" OR "Computational Intelligence" OR "Computer Vision Systems" OR "Knowledge Acquisition" OR "Machine learning" OR "Transfer Learning" OR "Large Language Model" OR "Adaptive Algorithm" OR "Trend Analysis" OR "Machine Pattern Recognition" OR "Machine Prediction Methods")) AND (((MH "medication adherence+") OR "medication adherence" OR "drug adherence" OR "prescription adherence" OR "medication persistence" OR "medication compliance" OR "drug compliance"))) AND (((MH "cardiovascular diseases+") OR "cardiovascular diseases" OR "adverse cardiac event" OR ((MH "coronary disease+") OR "coronary disease" OR "coronary heart disease") OR ((MH "cerebrovascular disorders+") OR "cerebrovascular disorders" OR "brain vascular disorders" OR "intracranial vascular disorders" OR "cerebrovascular insufficiency" OR "cerebrovascular occlusion") OR ((MH "peripheral arterial disease+") OR "peripheral arterial

disease") OR ((MH "rheumatic heart disease+") OR "rheumatic heart disease" OR "bouillaud disease") OR ((MH "heart defects, congenital+") OR "congenital heart disease" OR "abnormality heart" OR "malformation of heart") OR ((MH "venous thrombosis+") OR "deep vein thrombosis" OR ((MH "venous thrombosis+") OR (Venous AND Thrombosis) OR "venous thrombosis" OR phlebothrombosis) OR Phlebothromboses OR "thrombosis venous") OR ((MH "pulmonary embolism+") OR "pulmonary embolism" OR "pulmonary thromboembolisms")))

WoS: 13

(((ALL="artificial intelligence" OR ALL="Artificial Intelligence" OR ALL="Computer Reasoning" OR ALL="Machine Intelligence" OR ALL="Computational Intelligence" OR ALL="Computer Vision Systems" OR ALL="Knowledge Acquisition" OR ALL="Machine learning" OR ALL="Transfer Learning" OR ALL="Large Language Model" OR ALL="Adaptive Algorithm" OR ALL="Trend Analysis" OR ALL="Machine Pattern Recognition" OR ALL="Machine Prediction Methods")) AND ((ALL="medication adherence" OR ALL="medication adherence" OR ALL="drug adherence" OR ALL="prescription adherence" OR ALL="medication persistence" OR ALL="medication compliance" OR ALL="drug compliance"))) AND ((ALL="cardiovascular diseases" OR ALL="cardiovascular diseases" OR ALL="adverse cardiac event" OR (ALL="coronary disease" OR ALL="coronary disease" OR ALL="coronary heart disease") OR (ALL="cerebrovascular disorders" OR ALL="cerebrovascular disorders" OR ALL="brain vascular disorders" OR ALL="intracranial vascular disorders" OR ALL="cerebrovascular insufficiency" OR ALL="cerebrovascular occlusion") OR (ALL="peripheral arterial disease" OR ALL="peripheral arterial disease") OR (ALL="rheumatic heart disease" OR ALL="rheumatic heart disease" OR ALL="bouillaud disease") OR (ALL="heart defects, congenital" OR ALL="congenital heart disease" OR ALL="abnormality heart" OR ALL="malformation of heart") OR (ALL="venous thrombosis" OR ALL="deep vein thrombosis" OR (ALL="venous thrombosis" OR (ALL=Venous AND ALL=Thrombosis) OR ALL="venous thrombosis" OR ALL=phlebothrombosis) OR ALL=Phlebothromboses OR ALL="thrombosis venous") OR (ALL="pulmonary embolism" OR ALL="pulmonary embolism" OR ALL="pulmonary thromboembolisms")))
